# Demographics and Outcomes of Extracorporeal Membrane Oxygenation in COVID-19 Patients: National Database Analysis

**DOI:** 10.3390/jcm12186013

**Published:** 2023-09-16

**Authors:** Rami Ahmad, Andrew Abrahamian, Ayman Salih, Rayna Patel, Zachary Holtzapple, Ragheb Assaly, Fadi Safi

**Affiliations:** 1 Department of Internal Medicine, University of Toledo, Toledo, OH 43606, USAayman.salih@utoledo.edu (A.S.); rayna.patel@utoledo.edu (R.P.); zachary.holtzapple@utoledo.edu (Z.H.); ragheb.assaly@utoledo.edu (R.A.); fadi.safi@utoledo.edu (F.S.); 2 Department of Pulmonary and Critical Care, University of Toledo, OH 43606, USA

**Keywords:** COVID-19, extracorporeal membrane oxygenation, mortality, hospital stay, national inpatient sample

## Abstract

Introduction: The effectiveness of extracorporeal membrane oxygenation (ECMO) in treating COVID-19 patients has been variable. To gain a better insight, we examined the outcomes of ECMO in COVID-19 patients using data from the 2020 National Inpatient Sample database. Methods: We analyzed data from adult hospital admissions where COVID-19 was the primary diagnosis. The primary outcome was all-cause inpatient mortality. Secondary outcomes were length of stay (LOS), cost, and discharge disposition. Results: We identified 1,048,025 COVID-19 admissions, of which 98,528 were on mechanical ventilation (MV), and only 1.8% received ECMO. In-hospital mortality of mechanically ventilated patients who received ECMO was 49%, compared to 59% with no ECMO (*p* < 0.001). ECMO treatment was associated with a reduced risk of mortality (HR = 0.67, *p* < 0.0001, CI 0.57–0.79) even after adjustment for confounders and other comorbidities. Patients on ECMO had significantly extended hospital stays and were more likely to be discharged to an acute care facility. Younger and male patients were more likely to receive ECMO treatment. Females had a lower mortality risk, while race and obesity were not associated with an increased risk of death. Conclusion: ECMO treatment may offer survival benefits in severe COVID-19. Based on our findings, we suggest early ECMO treatment for patients with a high mortality risk.

## 1. Introduction

In 2019, the novel coronavirus infection (COVID-19) emerged as a global pandemic, affecting individuals with a wide range of symptoms, from asymptomatic and mild respiratory symptoms to more severe respiratory failure [1]. Almost 30% of COVID-19 patients admitted to the intensive care unit developed acute respiratory distress syndrome (ARDS) [2]. Extracorporeal membrane oxygenation (ECMO) is a salvage tool usually reserved for cases with severe hypoxic respiratory failure refractory to the conventional methods of mechanical ventilation [3]. 

Utilization of ECMO for COVID-19-related ARDS was based on pre-COVID-19 studies and experience from previous pandemics [4,5]. Early in the pandemic, studies reported a high mortality with the use of ECMO, leading investigators to recommend withholding ECMO initiation in COVID-19-related ARDS [6]. As the pandemic evolved, more studies revealed that the survival rate of patients receiving ECMO in COVID-19-related ARDS was similar to the survival rates of patients receiving ECMO for other causes of ARDS; most of these studies were retrospective, with variable sample sizes [5,7]. 

Due to the limited data on ECMO’s efficacy in patients hospitalized for COVID-19, our study uses 2020 Nationwide Inpatient Sample (NIS) data to determine the actual mortality of ECMO in COVID-19 patients and compares the outcomes of ECMO in COVID-19 and other primary diagnoses. This study sheds light on mortality outcomes, length of stay, and in-hospital complications. In addition, we investigate the impact of age, gender, race, and pre-existing comorbidities on the outcomes.

## 2. Methods

This retrospective observational cohort study used the Healthcare Cost and Utilization Project (HCUP) 2020 National Inpatient Sample (NIS) database. NIS is a publicly available inpatient database in the United States developed and maintained by the Agency for Healthcare Research and Quality, using data from the American Hospital Association’s yearly hospital survey. Data on patient demographics, diagnoses, and resources are gathered from a random 20% sample of all patients within each stratum, then collected and made available in the database. Each discharge is then weighted to represent the NIS nationally. We used the International Classification of Diseases, 10th revision, Clinical Modification (ICD-10-CM) coding system for each patient-level diagnosis code [8]. All ICD-10 codes we used to generate the primary and secondary diagnoses and the procedures codes are provided in the supplemental table. The study did not require institutional review broad approval since NIS uses publicly available de-identified data.

### 2.1. Study Population and Variables

We included all adult patients (age ≥ 18) admitted to the hospital with COVID-19 as the primary diagnosis. Patient demographics of age, gender, race, insurance, hospital size, and location were directly obtained from NIS data. We generated patient comorbidities of coronary artery disease, diabetes mellitus, chronic kidney disease, congestive heart failure, obesity, smoking status, and mechanical ventilation using ICD-10 codes. We identified ECMO using Veno-Venous (VV) or Veno-Arterial (VA) procedure codes. ECMO timing was calculated as days from the day of admission. The primary outcome was all-cause inpatient mortality. Secondary outcomes were the length of stay, discharge disposition (home, skilled nursing facility, short-term hospital, and home with home healthcare), and cost. We compared the demographics, comorbidities, and outcomes of patients on mechanical ventilation and who underwent ECMO to those of all patients with COVID-19 and to those of patients mechanically ventilated but who did not receive ECMO. In addition, we compared the outcome of ECMO in COVID-19 patients to those who underwent ECMO for other causes.

### 2.2. Statistical Analysis

Data analysis was performed using STATA (IC-17.0 version, STATA Corp, College Station, TX, USA). We used the chi-square test for categorical variables, Student’s *t*-test, and linear regression for continuous variables. Survival analysis was performed using the length of stay as the time variable and death as failure. We used univariable and multivariable Cox proportional hazards to calculate the unadjusted and adjusted odds of the primary outcome while accounting for potential confounders. All *p*-values were two-sided, and 0.05 was the threshold for statistical significance.

## 3. Results

We identified 1,048,025 hospital admissions with COVID-19 as the primary diagnosis. Ninety-eight thousand five hundred and twenty (98,520) of the admissions were on mechanical ventilation, and only 1.8% (1770) underwent ECMO while on mechanical ventilation. Patient demographics and comorbidities are described in Table 1. The mean age of the ECMO group was noticeably younger at 49.3 (95% CI 48.1–50.5), with a majority of the patients being male (69%). The most common race in all COVID-19 and ECMO groups was white. The Hispanic population constituted about 20% of all COVID-19 admissions; however, almost one-third (35%) of ECMO procedures were in admissions identified as Hispanic. Most ECMO patients were privately insured (52%), followed by Medicaid (25%). The majority of ECMO procedures were performed in teaching and large-sized hospitals, 80% and 94%, respectively. When comparing comorbidities, only obesity was significantly higher in the ECMO group, while all other comorbidities had a higher incidence in mechanical ventilation and all COVID-19 cases.

In-hospital mortality for COVID-19 patients on mechanical ventilation and who underwent ECMO was 49%, compared to 59% for MV patients without ECMO; both groups had significantly higher mortality than all COVID-19 patients (11%). The length of stay of ECMO patients was the highest at 34 (95% CI 31.4–37.2) days compared to 18.2 (95% CI 17.9–18.5) days in patients with mechanical ventilation only. For patients who received ECMO and survived hospital admission, around a quarter of them were discharged to a skilled nursing facility, while only 8% were discharged home. The average cost per admission in the ECMO group was considerably higher, with more than one million dollars per admission (Table 2).

The unadjusted hazard ratio of death during hospital admission was 0.49 (*p* < 0.001, CI 0.40–0.53); when controlling for age, race, sex, and other comorbidities, ECMO was still associated with a decreased hazard of death during hospitalization (HR = 0.67, *p* < 0.0001, 95% CI 0.57–0.79) (Table 3).

There was a total of 15,225 cases of ECMO in 2020. We compared the outcome of ECMO in COVID-19 and in other indications. The mortality rate of the two groups was comparable but slightly higher in the COVID-19 group (49% vs. 41%, *p* = 0.0013), with a more extended hospital stay of 33.6 days (95% CI 30.8–36.5) vs. 29.5 days (95% CI 27.7–31.4) (*p* = 0.014). The average time to ECMO was 6.9 days (95% CI 5.8–8.0) in COVID-19 patients, slightly longer than in the other group (5.6 (95% CI 4.9–6.3) days). Regarding ECMO complications, the COVID-19 group experienced a higher incidence of sepsis but no statistically significant difference in shock or blood product transfusion requirement (Table 4). Table 5 demonstrates the most common primary diagnoses in admissions who underwent ECMO: sepsis (15%), COVID-19 (13%), and myocardial infarction (6.5%).

## 4. Discussion

### 4.1. Primary Analysis

In this large database analysis, we analyzed the outcome of ECMO in patients affected with COVID-19 during the first year of the pandemic, 2020, in the United States. Our analysis showed that ECMO patients who required mechanical ventilation secondary to COVID-19 infection had a lower mortality rate, which is consistent with other studies on ECMO outcomes in ARDS secondary to COVID-19. The two main randomized controlled trials that compared outcomes of ECMO in patients with severe ARDS versus conventional mechanical ventilation were the CESAR and EOLIA trials. Both trials and the subsequent meta-analysis demonstrated survival benefits and improved mortality of ECMO in patients with ARDS [3,9,10,11]. 

The mortality rate in our cohort was consistent with previous reports of ECMO mortality in COVID-19 patients. Studies from different parts of the world reported mortality rates ranging from 31% to 68% [5,12,13,14]. The Extracorporeal Life Support Organization (ELSO) registry data from the first year of the pandemic found a mortality range of 36.9% in the early months, which then increased to 58% later in 2020. This difference in mortality could be explained by the difference in patient selection criteria or differences between the centers where ECMO was performed. As the pandemic progressed, higher ECMO mortality rates were likely the result of ECMO being used exclusively in treatment-resistant patients; as patients later on in 2020 were more likely to be treated with corticosteroids, remdesivir, and non-invasive ventilation pre-intubation [15]. When comparing ECMO directly to mechanical ventilation, retrospective and observational prospective studies conducted in both the United States and Europe demonstrated a significant benefit in mortality [5,16,17]. A report by the COVID-19 Critical Care Consortium found a 7.1% decrease in 60-day mortality risks in patients receiving ECMO [17], slightly less than the 10% decrease in mortality in our analysis. 

Despite the benefits of ECMO, it comes with a high cost and strains on the healthcare system. The LOS of ECMO admissions was considerably higher than that of the conventional mechanical ventilation group; however, it was very close to the LOS reported in the CESAR trial, at 35 days [9]. Hence, a prolonged LOS did translate into a tremendously higher average cost per admission. In addition to hospital costs, only 12% of the patients who survived ECMO were discharged home with or without healthcare, while the rest were discharged to acute hospitals or skilled nursing facilities. COVID-19 patients who undergo ECMO and prolonged mechanical ventilation are associated with muscle wasting and weakness [18,19], which would explain the need for rehabilitation after discharge. Another factor influencing discharge disposition was primary insurance, since the majority of ECMO patients were privately insured or had Medicaid; this would expectedly result in ECMO patients having more access to acute rehab facilitates upon discharge and subsequently an increased cost and utilization of resources. 

### 4.2. Secondary Analysis

Our analysis of the demographic characteristics of NIS patients observed a majority of younger males in the ECMO group, compared to the other groups. This is not unexpected, as it has already been established that ECMO’s ability to improve survival decreases with age; it would be reasonable for providers to direct younger patients to more invasive therapy, especially in a pandemic setting with limited resources [9,20]. Male patients being more predisposed to ECMO would coincide with the male gender as an independent risk factor for COVID-19 severity [21,22]. The disparity in gender survival corresponds with our finding that being female had a protective effect on mortality, with a hazard ratio of 0.91 (95% CI 0.87–0.94). 

Race, in general, was not associated with a statistically significant mortality risk. There are mixed reports in the literature on the impact of race on disease severity, admission to hospital, and survival [23,24]. Hispanics were overly represented in ECMO patients at 34%, while they only constituted 20% of all admitted COVID-19 patients. There are several reports about racial disparities among Hispanic minorities, due to socioeconomic status and limited access to healthcare, which could lead to increased disease severity [25]. 

The ECMO group had a lower incidence of all co-morbidities except for obesity, which could be explained by their relatively younger age and fewer chronic diseases. Obesity has been associated with an increased risk of hospitalization and death from COVID-19 [26], and it would be anticipated that obese patients meeting the criteria for ECMO would have disproportionally higher mortality rates. Historically, obesity was considered a relative contraindication for ECMO [27]. Many studies have showed excess mortality of ECMO for patients with a high BMI [28]. A propensity score matching analysis of more than 18,000 by the ELSO group found that obesity, defined as BMI ≥ 35, was associated with lower in-hospital mortality [29]. Our data are consistent with the reported literature and illustrate that obesity should not be considered a contraindication for ECMO. 

## 5. Limitations

Our study has a few limitations. First, this study was not a randomized control trial but a retrospective cohort study in which we could not control for residual confounders. Second, NIS data do not provide specific but essential details about medical management, such as ventilator settings or medications. Such information may have provided us with more precise conclusions and more specific recommendations concerning patient care. NIS data also do not provide the specific criteria or clinical indications for ECMO besides the need for ventilatory support. Third, NIS data are derived from medical coding rather than directly obtained from reviewing the charts and documentation of medical providers. Any inaccuracies or limitations in coding would have constrained our results. Despite these limitations, we expect our study’s significant size and scope, with over one million hospital admissions from across the United States, to correct for any of the limitations described above and create concrete evidence.

## 6. Conclusions

Our research has shown that COVID-19 patients who received ECMO treatment while on mechanical ventilation had a reduced risk of death while in the hospital. Age and being male were linked to a higher risk of mortality, but race did not appear to have an impact. The effect of medical comorbidities on mortality varied. Our findings suggest that patients with COVID-19-related ARDS who are at high risk should be considered for ECMO treatment promptly to increase their chances of survival.

## Figures and Tables

**Table 1:** Patient demographics and characteristics.

	MV ECMO (*n* = 1770)	MV No ECMO (*n* = 98,520)	All COVID-19 (*n* = 1,048,025)
**Age** (95% CI)	49.3 (48.1–50.5)	65.1 (64.9–65.3)	64.7 (64.6–64.9)
**Sex** (%)			
*Male*	1215 (68.6%)	59,112 (60%)	553,090 (53%)
*Female*	555 (31.3%)	39,408 (40%)	494,935 (47%)
**Race**			
*White*	580 (35.1%)	43,180 (46.2%)	535,620 (52.7%)
*Black*	350 (20%)	18,420 (19.7%)	187,670 (18.5%)
*Hispanic*	580 (35%)	22,420 (24%)	208,400 (20.5%)
*Asian*	43 (2.4%)	3380 (3.5%)	32,775 (3.2%)
*Native*	40 (2.3%)	1580 (1.7%)	10,555 (1%)
*Others*	69 (3.9%)	4505 (4.8%)	41,535 (4.1%)
**Comorbidities**			
*CAD*	65 (3.7%)	19,040 (19.6%)	192,815 (18.3%)
*CHF*	290 (16.3%)	21,185 (21.9%)	166,940 (15.9%)
*CPD*	335 (18.9%)	25,340 (26.1%)	251,455 (23.9%)
*DM*	590 (33.3%)	48,010 (49.6%)	420,050 (40%)
*CKD*	145 (8.2%)	24,485 (25.3%)	207,975 (19.8%)
*Smoking*	220 (12.4%)	22,215 (22.9%)	283,155 (27%)
*Obesity*	780 (44.1%)	28,210 (29.2%)	247,285 (23.6%)
**Insurance**			
*Medicare*	155 (9%)	51,835 (54%)	548,375 (52%)
*Medicaid*	445 (25%)	12,860 (13.3%)	121,230 (12%)
*Private*	920 (52%)	23,785 (25%)	289,420 (30%)
*Self-Pay*	85 (4.8%)	3030 (3.1%)	35,705 (3%)
*Other*	160 (9%)	4805 (5%)	48,675 (5%)
**Hospital Size**			
*Small*	105 (6%)	21,645 (22.4%)	269,735 (25.7%)
*Medium*	235 (13.3%)	27,925 (28.9%)	303,300 (28.9%)
*Large*	1430 (81%)	47,180 (48%)	474,990 (45.3%)
**Hospital Location**			
*Rural*	10 (0.5%)	8945 (9.2%)	123,315 (11.7%)
*Urban Nonteaching*	90 (5.1%)	16,235 (16.7%)	202,585 (19.3%)
*Urban Teaching*	1670 (94%)	71,570 (74%)	722,125 (69%)

MV: mechanical ventilation, CAD: coronary artery disease, CHF: congestive heart failure, CPD: chronic pulmonary diseases, DM: diabetes mellitus, CKD: chronic kidney disease.

**Table 2:** Mortality and morbidity of ECMO, mechanical ventilation, and all COVID-19 patients.

	ECMO	MV	All COVID	*p* Value
**Mortality**	860 (48.6%)	57,280 (59.2%)	117,240 (11%)	<0.001
**Length of Stay** (Days) (95% CI)	34 (31.4–37.2)	18.2 (17.9–18.5)	7.42 (7.36–7.49)	<0.001
**Discharge Disposition**				<0.001
*Home*	140 (7.9%)	7744 (7.9%)	572,536 (54.6%)	
*Short-term hospital*	280 (15.8%)	8217 (8.3)	30,917 (2.9%)	
*Skilled Nursing Facility*	415 (23.4%)	17,763 (18%)	175,754 (16.8%)	
*Home With Home Healthcare*	75 (4%)	6088 (6.2%)	140,750 (13.4%)	
**Cost** (Average per admission)	1,118,644	275,071	78,601	<0.0001

**Table 3:** Cox proportional hazard of mortality during hospital admission.

	HR	Standard Error	t	*p* > t	95% CI
**Female**	0.91	0.018	−4.96	<0.001	0.87–0.94
**Age**	1.02	0.001	27	<0.001	1.02–1.03
**Race**	0.98	0.009	−1.6	0.11	0.97–1.00
**ECMO**	0.69	0.058	−4.32	<0.0001	0.59–0.82
**CHF**	1.07	0.025	2.72	0.007	1.02–1.12
**CKD**	1.24	0.029	9.26	<0.001	1.18–1.30
**CAD**	1.22	0.028	8.4	<0.001	1.16–1.27
**CPD**	0.99	0.021	−0.19	0.85	0.95–1.04
**DM**	0.99	0.020	−0.25	0.81	0.96–1.03
**Obesity**	0.98	0.022	−0.92	0.36	0.94–1.02
**Smoking**	1.08	0.026	3.08	0.002	1.03–1.13

CAD: coronary artery disease, CHF: congestive heart failure, CPD: chronic pulmonary diseases, DM: diabetes mellitus, CKD: chronic kidney disease.

**Table 4:** ECMO outcomes in COVID-19 and non-COVID-19 patients.

	ECMO No-COVID	ECMO-COVID	*p* Value
**Inpatient Mortality**	5375 (41%)	995 (49%)	0.0013
**Length of Stay** (days) (95% CI)	29.5 (27.7–31.4)	33.6 (30.8–36.5)	0.014
**Time to ECMO** (days) (95% CI)	5.6 (4.9–6.3)	6.9 (5.8–8.0)	0.053
**Complications**			
AKI	9109 (69%)	1259 (62.4%)	0.018
Sepsis	2915 (22%)	1055 (52%)	<0.001
Shock	8685 (65.7%)	1360 (67.3%)	0.60
Anemia	6020 (45.6%)	735 (36.4%)	0.005
Blood Product Transfusion	3665 (27.6%)	625 (31%)	0.26

**Table 5:** Most common primary diagnosis in patients who underwent ECMO.

Most Common Primary Diagnosis in ECMO	Percentage
Sepsis	2355 (15%)
2. COVID-19	2050 (13%)
3. Myocardial Infarction	975 (6.4%)
4. Acute Hypoxic Respiratory Failure	360 (2.4)
5. Idiopathic Pulmonary Fibrosis	310 (2.0)
6. Acute Respiratory Distress Syndrome (ARDS)	295 (1.9%)

## Data Availability

NIS data can be accessed through the following link https://hcup-us.ahrq.gov/nisoverview.jsp.

## Supplementary Materials

The following supporting information can be downloaded at: https://www.mdpi.com/article/10.3390/jcm12186013/s1.
