# Supplementary material for: Demographics and Outcomes of Extracorporeal Membrane Oxygenation in COVID-19 Patients: National Database Analysis
Source: J Clin Med. 2023 Sep 16;12(18):6013. doi: 10.3390/jcm12186013 (PMC10532225; doi:10.3390/jcm12186013)
Supplement: Supplementary file 1 [file jcm-12-06013-s001.zip › jcm-2574008-supplementary.pdf]

Supplemental table S1: ICD-10 Codes used to generate analysis.

**Variable**	**Codes**
COVID	U071
ECMO	5A1522H, 5A1522G, 5A15A2G, 5A15A2H, 5A152, 5A15A
Mechanical Ventilation	5A1935Z, 5A1945Z, 5A1955Z
CHF	I50, I110, I130
CPD	J40, J41, J42, J43, J44, J45, J47, J84
Smoking	F172, Z720, Z87891
DM	E11
CKD	N18
Obesity	Z683, Z6841, Z6842, Z6843, Z6844, Z6845
CAD	I25
Sepsis	A41
Shock	R57, R6521
Blood transfusion	302
